# Does algae β-glucan affect the fecal bacteriome in dairy calves?

**DOI:** 10.1371/journal.pone.0258069

**Published:** 2021-09-30

**Authors:** Gercino Ferreira Virginio Junior, Maria Eduarda Reis, Ana Paula da Silva, Ariany Faria de Toledo, Amanda Moelemberg Cezar, Lucas William Mendes, Leandro Greco, Horácio Montenegro, Luiz Lehmann Coutinho, Carla Maris Machado Bittar

**Affiliations:** 1 Department of Animal Science, “Luiz de Queiroz” College of Agriculture, University of Sao Paulo, Piracicaba, São Paulo, Brazil; 2 Center for Nuclear Energy in Agriculture, University of Sao Paulo, Piracicaba, São Paulo, Brazil; 3 Kemin Animal Nutrition and Health Division South America, Valinhos, São Paulo, Brazil; University of Illinois, UNITED STATES

## Abstract

β-glucans has been reported to be associated with many health-promoting and improvements in animal performance, however, information about their effects on the bacterial community remains unknown. This study aimed to investigate how the addition of β-glucans can affect the fecal bacterial community with possible consequences on animal growth and health. For this, newborn Holstein calves (n = 14) were individually housed in tropical shelters and blocked according to sex, date, and weight at birth and randomly assigned to one of the following treatments: (1) Control: milk replacer (14% solids, 24% CP, 18.5% fat); (2) β-glucans: milk replacer supplemented with β-glucans (2 g/d). All calves were bucket fed 6 L/d of milk replacer and received water and starter concentrate ad libitum starting on d 2. To evaluate the bacteriome, fecal samples were collected at weeks 1, 2, 4, and 8. The bacterial community was assessed through sequencing of the V3-V4 region of the 16S rRNA gene on the Illumina MiSeq platform and analyzed using the DADA2 pipeline. No differences for Shannon and Chao1 indexes were observed for treatments, but both indexes increased with age (P < 0.001). There were dissimilarities in the structure of the bacterial community during the pre-weaning period (*P* = 0.01). In a deeper taxonomic level, *Collinsella* (Actinobacteriota), *Prevotella* (Bacteroidota), and *Lactobacillus* (Firmicutes) were the most abundant genera (9.84, 9.54, and 8.82% of the sequences, respectively). β-glucans promoted a higher abundance of *Alloprevotella* and *Holdemanella*, which may indicate a beneficial effect of supplementation on dairy calves. The bacterial community was highly correlated with the fecal score at weeks 1 and 2 and with starter concentrate intake at week 8. In conclusion, algae β-glucan supplementation could be beneficial to fecal bacteriome and consequently to the health and performance of dairy calves.

## Introduction

β-glucans (BG) are polysaccharides found in the cell wall of cereals, algae, yeasts, or bacteria, and their use in human and animal nutrition is associated with improved immune function [1–3]. The BGs are also considered natural prebiotics and have several biological functions, such as competing against pathogenic microorganisms for binding sites on the intestinal epithelium [4], as well as preventing inflammatory processes [5]. Unlike probiotics, few studies have been conducted to understand the effects of prebiotics on ruminants [3]. However, in nonruminants and humans, the effects are beneficial, such as promoting higher bacterial richness, higher abundance of *Bifidobacterium* and *Lactobacillus*, immunomodulation, nutrient absorption effects, and an improved gut barrier [6–9].

In ruminants, prebiotics are commonly used in dairy calf feeding, and oligosaccharides are the main molecule used [3]. Studies evaluating the starter concentrate of BG supplementation have observed specific results, such as stimulation of the immune system, a greater abundance of *Lactobacillus* and fibrolytic bacteria, and a lower abundance of methanogenic archaea [10, 11]. In this type of supplementation, BGs are metabolized directly in the rumen-reticulum. However, Ma et al. [12] supplied BG in milk replacer and observed a higher nutrient digestibility, higher villous height to crypt depth ratio, as well as a greater effect on the immune system, such as a higher proportion of immunoglobulins, IgG, and IgM, and also lysozyme and alkaline phosphatase. Supplementation through the milk replacer allows the BG to act on the intestinal microbiota since the esophageal groove the milk replacer flows directly into the abomasum, and the BG will be metabolized at the intestinal level, as occurs in nonruminants animals. Nevertheless, the effect of BG on the gut microbiota of calves has not yet been studied.

The gut microbiota plays a fundamental role in the development and function of the gastrointestinal tract and gut health [13], and a better understanding of the effects of prebiotics on the gut microbial communities is of paramount importance. A practical, non-invasive way to collect samples over time to assess the gut microbiota in calves is fecal sampling [14, 15]. However, this sampling is more accepted as a proxy for the distal gut microbiota, mainly because of the differences in the bacterial community along the intestinal tract [16].

This study aimed to investigate if algae β-glucan supplementation affects the fecal bacteriome of dairy calves in the pre-weaning phase. For this, we have assessed the fecal bacterial community of dairy calves using the 16S rRNA gene amplicon sequencing as a non-invasive proxy for the intestinal bacterial community in fourteen young dairy calves. We hypothesized that algae β-glucan supplementation on the milk replacer can increase the abundance of beneficial groups in the fecal bacterial community during the pre-weaning period in dairy calves.

## Material and methods

### Animals, facilities, and experimental design

This study was conducted at the experimental calf facilities of the Department of Animal Science, Luiz de Queiroz College of Agriculture, University of São Paulo, located in Piracicaba—São Paulo, Brazil. All animal procedures followed the guidelines recommended by the institutional Animal Research Ethics (Protocol n°. 2019–11).

This study was part of a performance study with 34 newborn Holstein calves, blocked according to sex, age, and birth weight (36.38 ± 1.3 kg) and evaluated in a randomized block design during the pre-weaning period for performance and health. Previous performance results were published in the abstract by Reis et al. [17]. For the present study, 14 calves were assigned to seven blocks, where each block had two calves with similar sex, age, and birth weight (35.26 ± 1.4 kg) at the beginning of the experiment and were used to assess the impact of β-glucans on the fecal bacterial community.

All calves were fed 10% of birth-weight of high-quality colostrum (> 50g IgG/L) within the first 6 hours of life [18], and all calves had serum protein above 5.5 g/dL at 48 hours of life. The calves were randomly assigned to receive either, Control: a commercial milk replacer (MR; nothing added); or BG: the same commercial MR supplemented with algae β-glucans (2g/d Aleta^™^, Kemin Industries, Inc., USA).

### Housing and feeding management

All calves were housed in individual wood shelters (1.35 m in height, 1 m in width, and 1.45 m in depth) with buckets for feed and water. The wood shelters were distributed in a trimmed grassy field. The animals were bucket-fed with 6 L of a commercial MR reconstituted to 14% solids (Sprayfo Azul, Sloten do Brazil Ltd., Santos, SP, Brazil; Table 1), split into two meals (0700 h and 1700 h).

**Table 1:** Chemical composition of calf starter and milk replacer.

Item, g/Kg	Calf starter^1^	Milk replacer ^2^
DM	893.0	961.0
Ash	96.0	88.0
Crude protein	247.0	224.0
Crude fat	52.0	162.0
NDF^3^	138.9	0.6
ADF^4^	55.0	-
NFC^5^	466.0	522.0

^1^Commercial calf starter (Ração Bezerra AgMilk Agroceres Multimix Nutrição Animal Ltda., Rio Claro, SP, Brazil).

^2^Milk replacer (Sprayfo Azul, Sloten do Brazil Ltd., Santos, SP, Brazil) was fed to both treatments diluted to 14% of solids.

^3^NDF, neutral detergent fiber;

^4^ADF, acid detergent fiber;

^5^NFC, non-fiber carbohydrates.

A precision scale (AUY220, Shimadzu Corporation, Kyoto, Japan) was used to weigh the amount of BG per feeding. Every day, 2 g of BG (1 g/meal) was added and sufficiently mixed to the diluted MR (3 L/meal) just before feeding. Water and a commercial calf starter (Table 1) were available for ad libitum intake throughout the 56-d study. The starter was offered every morning, just after milk feeding, and was available until the following morning, when orts were weighted for daily intake calculations. Calves were enrolled in this study for the preweaning period, d 1 to d 56. Calves were gradually weaned after the trial ended.

### Animal health and performance measurements

The individual liquid diet and starter concentrate intakes were measured daily. Calves were weighed at birth and weekly until week 8 on a mechanical scale (ICS-300, Coimma Ltda., Dracena, SP, Brazil), always before morning feeding. Average daily gain (ADG) was calculated for the pre-weaning period.

Fecal consistency was scored on a scale of 0 to 3, where the fecal score of 0 = normal consistency, 1 = semiformed or pasty, 2 = loose feces, and 3 = watery feces (McGuirk, 2008). A fecal score ≥ 2 was considered a diarrhea bout when it occurred for more than 2 consecutive days [19]. Calves with a score ≥ 2 were bottle-fed an oral rehydration solution 2 h after morning feeding, until the fecal score returned to 1 or 0. The calves’ rectal temperature was measured daily using a digital thermometer, and fever was considered when it was above 39.4 °C. Health problems were monitored and treated according to veterinary recommendations.

### Blood sampling and analysis

Blood samples were collected weekly, 2 h after the morning milk feeding via jugular vein puncture with evacuated tubes (Vacutainer, Becton Dickinson, Franklin Lakes, NJ). The samples were collected in two different tubes containing either sodium fluoride as an anti-glycolytic and potassium EDTA as an anticoagulant to obtain plasma, or a clot activator to obtain serum. After collection, the samples were centrifuged at 2,000 × g for 20 minutes at 4 °C to obtain plasma and serum. The determination of selected blood metabolites was performed on an Automatic Biochemistry System—Model SBA—200 (CELM, Barueri, SP, Brazil) using commercial kits (LABTEST Diagnóstica S.A., Lagoa Santa, MG, Brazil). The selected metabolites, protein [albumin (Ref. 19), creatinine (Ref. 35), total serum protein (Ref. 99)], carbohydrates [glucose (Ref. 85)], and urea (Ref. 104) were chosen to study the effects of β-glucans in the intermediate metabolism.

### Evaluation of bacterial community

#### Fecal samples collections

Fecal samples were collected at days 7 (1st week), 14 (2nd week), 28 (4th week), and 56 (8th week, weaning). The samples were collected manually with gloves, directly from the animals’ rectum, and the gloves were discarded at each collection to avoid cross-contamination among samples. About 2 g of feces were placed in sterile tubes, and immediately frozen at -20 °C.

#### DNA extraction, library preparation, and sequencing

DNA extraction from fecal samples was performed using the QIAamp^®^ Fast DNA Stool Minikit extraction (Qiagen, Hilden, Germany), following the modifications suggested by Yu and Morrison [20]. The quality of the DNA samples was evaluated by electrophoresis on 0.8% agarose gel and concentrations were quantified with a spectrophotometer NanoDrop^®^ ND-2000 (Thermo Fisher Scientific, Wilmington, DE, USA).

For taxonomical profiling, amplicon-sequencing targeting the V3–V4 region of the 16S rRNA gene of Bacteria and Archaea were performed at the Center for Functional Genomic Research (ESALQ/ USP), Piracicaba, Brazil. The amplicon libraries were prepared using the Miseq Reagent Kit v3 (Illumina, San Diego, CA, USA), following the manufacturer’s instructions for the Illumina MiSeq platform (2 × 250 bp paired-end). The full-length primers sequence were:16S Amplicon PCR Forward Primer: 5’ TCGTCGGCAGCGTCAGATGTGTATAAGAGACAGCCTACGGGNGGCWGCAG; 16S Amplicon PCR Reverse Primer: 5’ GTCTCGTGGGCTCGGAGATGTGTATAAGAGACAGGACTACHVGGGTATCTAATCC.

All raw DNA sequence reads were deposited in NCBI’s Sequence Read Archive under BioProject PRJNA717280, submission SUB9353840.

#### Bioinformatic analyses

The bioinformatics analyses were conducted similarly to the study of Virgínio Júnior et al. [15]. The data were analyzed using a set of packages implemented in the R language (R Core Team) and available through the BioConductor project [21–23].

The multiplexed readings were assigned to biological samples. The DADA2 program [24] was used to model and correct amplicon errors without building OTUs. The filtering of fastq files was performed to cut the PCR primers’ sequences and filter the 3’ends of the readings due to the quality decay (Q < 30) but maintaining the overlap for later joining of the readings and reassembly of the fragment of the V3-V4 region. The DADA2 algorithm uses a parametric error model, and each set of amplicon data has a different set of error rates.

After the initial processing of the sequencing data by DADA2, taxonomies were assigned to each ASV (Amplicon Sequencing Variants) using an implementation of the DADA2 program of the naive Bayesian classifier method [25]. The ‘assignTaxonomy’ function takes as input a set of sequences (ASVs) to be classified, and a set of training of reference sequences with known taxonomy and assigns taxonomies. The SILVA database v138 was used as a reference [26].

The taxonomic classifications generated by DADA2, and their quantifications, were imported into the phyloseq program [27], also implemented in R. The α and β diversity analyses were performed with the phyloseq package, as described in Callahan et al. [23]. For the β-diversity analysis, a multivariate permutational analysis of variance (PERMANOVA) was performed, using weighted UniFrac distances, testing the treatment effect, week, and interaction. ASVs that have not been classified until the family level were filtered, and ASVs marked as being the same species have been clustered. After applying these filters, the tables of gross abundance and relative abundance counts were obtained.

Then, the taxonomic counts in the phyloseq object were imported into the edgeR package [28] to normalize the sizes of each sample’s libraries [29], subsequently, the counts were transformed to the base 2 logarithms of the counts per million (log CPM) of each sample (voom transformation) [30]. These transformations allow the linear models implemented in the limma package [31] to analyze differential abundance. Finally, after adjusting the linear model with limma, the differential taxonomic abundance was tested for each contrast (pair of treatments) with moderate t-tests [32].

To evaluate the bacterial community structure and correlate it with the animal performance (starter concentrate intake and ADG), blood metabolites (TSP, Creatinine, and Glucose) and health (Fecal score and rectal temperature) data we used Canonical correspondence analysis (CCA). To verify the significance of the calves’ parameters upon the microbial community we used Forward Selection (FS) followed by the Monte Carlo permutation test with 1,000 random permutations. To test whether the sample categories harbor significantly different bacterial communities we used the PERMANOVA [33]. To further explore the relationship between the relative abundance of microbial groups at the phylum and genus levels with the animal performance and health data, we calculated Spearman’s rank correlation coefficients using the ‘multtest’ package in R, and the correction was made using Benjamini-Hochberg FDR. For visualization, a heatmap was constructed using the R package ‘corrplot’.

## Results

Sequencing information (Number of raw reads, number of quality-filtered reads, Number ASVs identified) is shown in S1 and S2 Tables.

**Fig 1:**
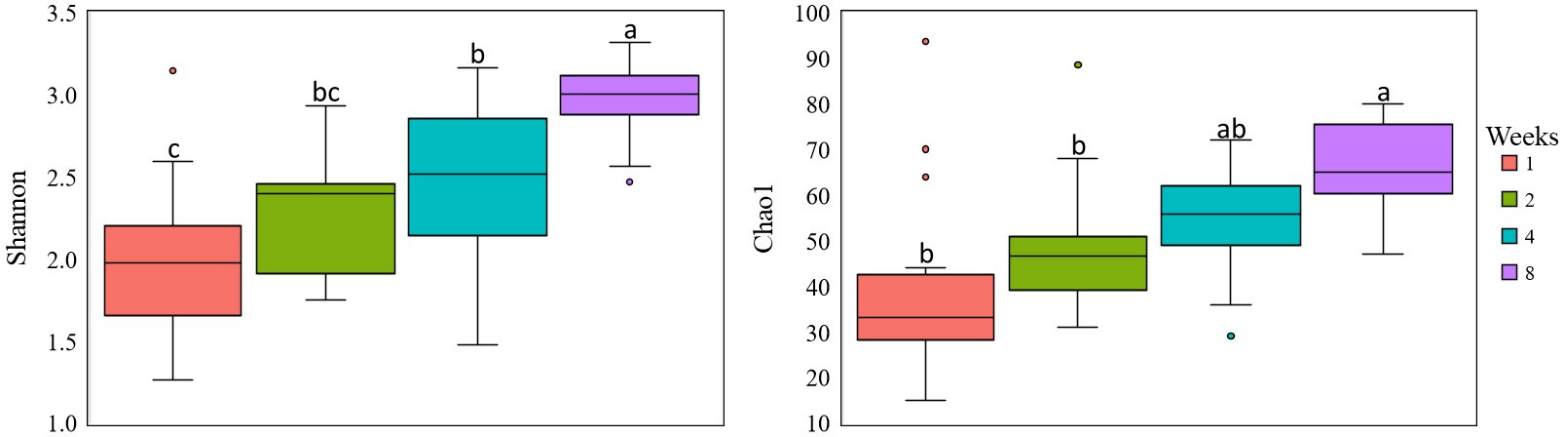
α-diversity in dairy calves’ feces fed milk replacer supplemented or not with β-glucans. Letters above boxes indicate significant differences at *P* < 0.05. Data are visualized as box-plots showing the median and the interquartile (midspread) range (boxes containing 50% of all values), the whiskers (representing the 25 and 75 percentiles) and the extreme data points.

The analysis of α-diversity showed that diversity increased with age (Fig 1) for both Shannon (Fig 1A; *P* < 0.001) and Chao1 indexes (Fig 1B; *P* < 0.001). No differences for Shannon and Chao1 were found between treatments or interactions for treatment and age.

**Fig 2:**
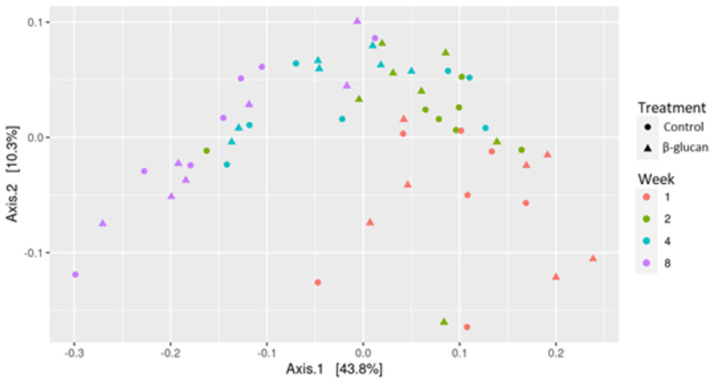
β-diversity of bacterial community in dairy calves’ feces fed milk replacer supplemented or not with β-glucans. Multidimensional scaling (MDS) showing the weighted UniFrac distance metric.

The same effect was observed for β-diversity (Fig 2). Structurally, supplementation or the interactions between supplementation and age have no effect, but there are dissimilarities among weeks (PERMANOVA, *P* < 0.001). The two main axes of the MDS display 54.1% of the data variation in the composition of the fecal bacterial community.

**Fig 3:**
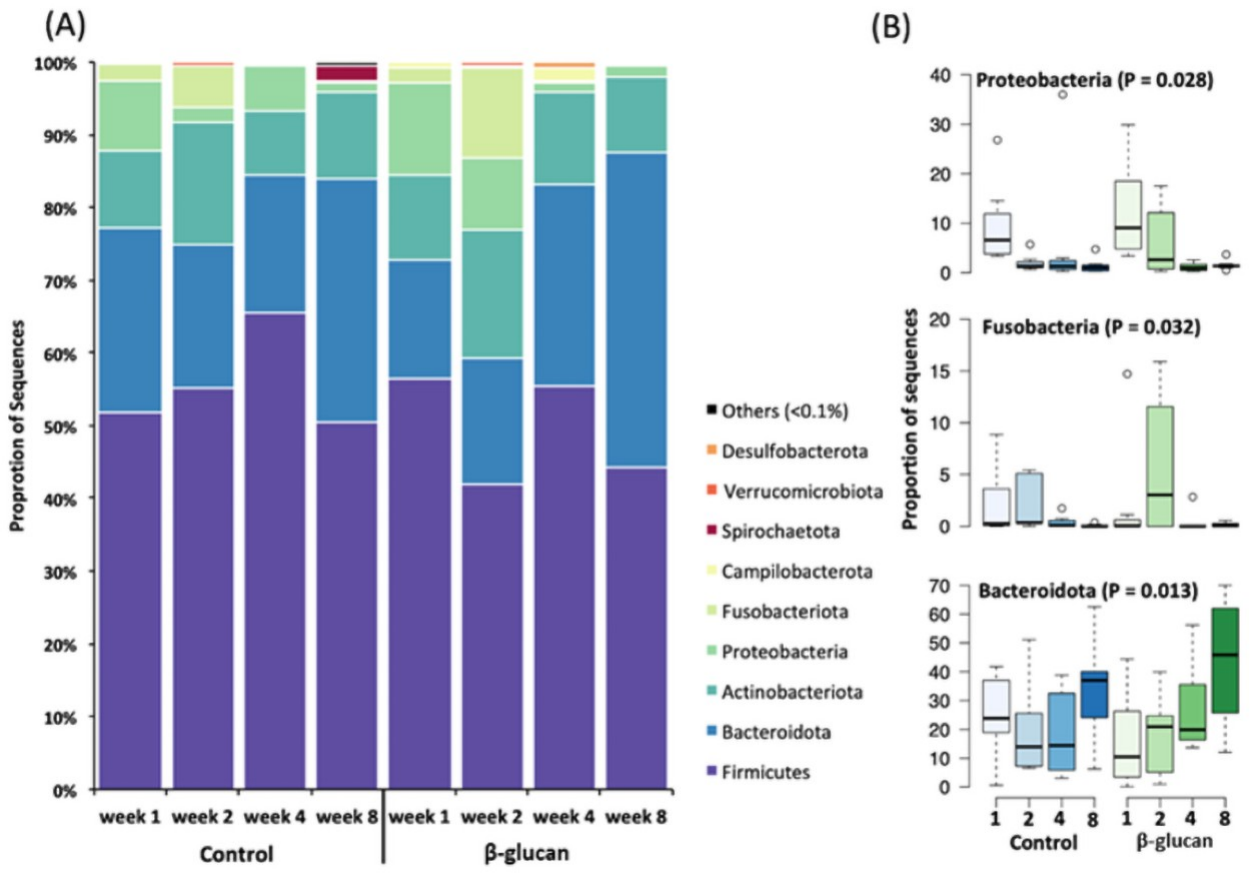
Bacterial composition in dairy calves’ feces fed milk replacer supplemented or not with β-glucans. (a) Phyla composition and (b) differential abundance over the weeks.

Overall, fecal samples were dominated by the phylum Firmicutes (52.62% of the total sequences), followed by Bacteroidota (25.26%), Actinobacteriota (12.58%), Proteobacteria (5.57%), and Fusobacteriota (3.00%; Fig 3A), which together accounted for more than 90% of the sequences (Fig 3). Other phyla with less than 1% of sequence abundance are described in S3 Table. Supplementation had no effects on phyla composition, but for age effect only three phyla were statistically different. Proteobacteria and Fusobacteria decreased in abundance until weaning (*P* = 0.028; *P* = 0.033, respectively), while Bacteroidota increased (*P* = 0.013; Fig 3B).

**Fig 4:**
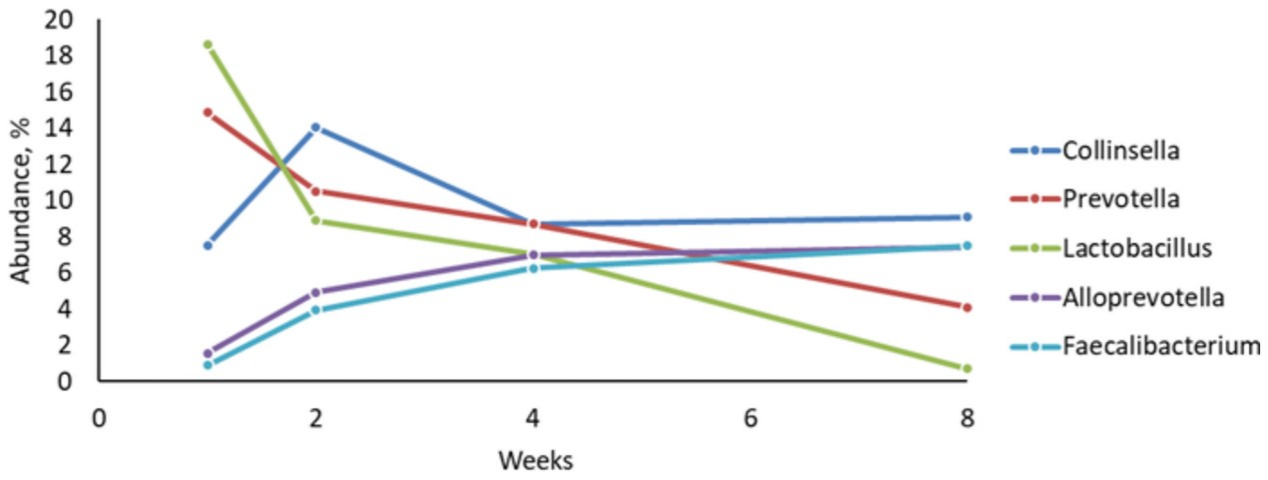
Changes in relative abundance of dominant bacterial genera over time in dairy calves’ feces fed milk replacer supplemented or not with β-glucans.

At the genus level, fecal samples were dominated by *Collinsella* (9.84%), *Prevotella* (9.54%), *Lactobacillus* (8.82%), *Alloprevotella* (5.23%), and *Faecalibacterium* (4.65%). Altogether, 261 genera were identified and 43 were unclassified, corresponding to 12.12% of the total abundance (S4 Table). *Lactobacillus* and *Prevotella* were the most abundant at week 1 (18.62 and 14.85%, respectively), but their abundance decreased until week 8 (0.72 and 4.10%, respectively; Fig 4). *Collinsella* was the most abundant between weeks 2 and 8. Both genera *Alloprevotella* and *Faecalibacterium* increased in abundance until week 8 (Fig 4).

**Fig 5:**
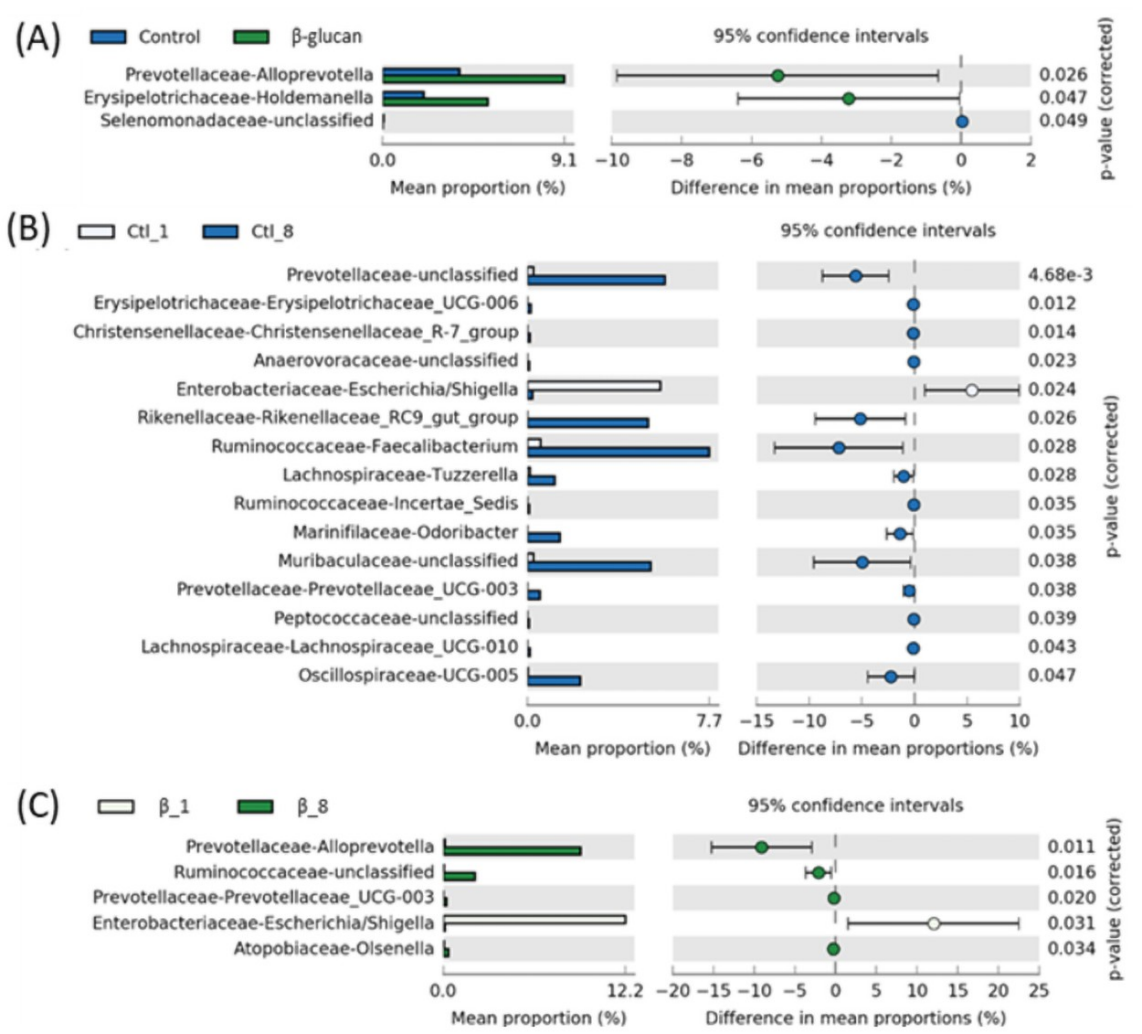
Bacterial genera composition in dairy calves’ feces fed milk replacer supplemented or not with β-glucans. Scatter-plot based on Welch’s t-test with Benjamini–Hochberg correction constructed in STAMP (P < 0.05). (A) differences between treatments; (B) differences between week 1 and week 8 in control group (Ctl), and (C) differences between week 1 and week 8 in β-glucans supplemented group.

Three genera were affected by BG supplementation (Fig 5). The lower abundance of an unclassified Selenomonadaceae (*P* = 0.049) and higher abundance of *Alloprevotella* and *Holdemanella* were observed for β-glucans supplemented animals compared to control (*P* = 0.026 and *P* = 0.046, respectively; Fig 5A). As the major difference was among weeks, Fig 5B shows the difference between weeks 1 and 8 for control animals (15 genera), and Fig 5C for β-glucans animals (5 genera). Only the *Escherichia/Shigella* genus had higher abundance at week 1 for both control and β-glucans animals (*P* = 0.024; *P* = 0.031, respectively).

**Fig 6:**
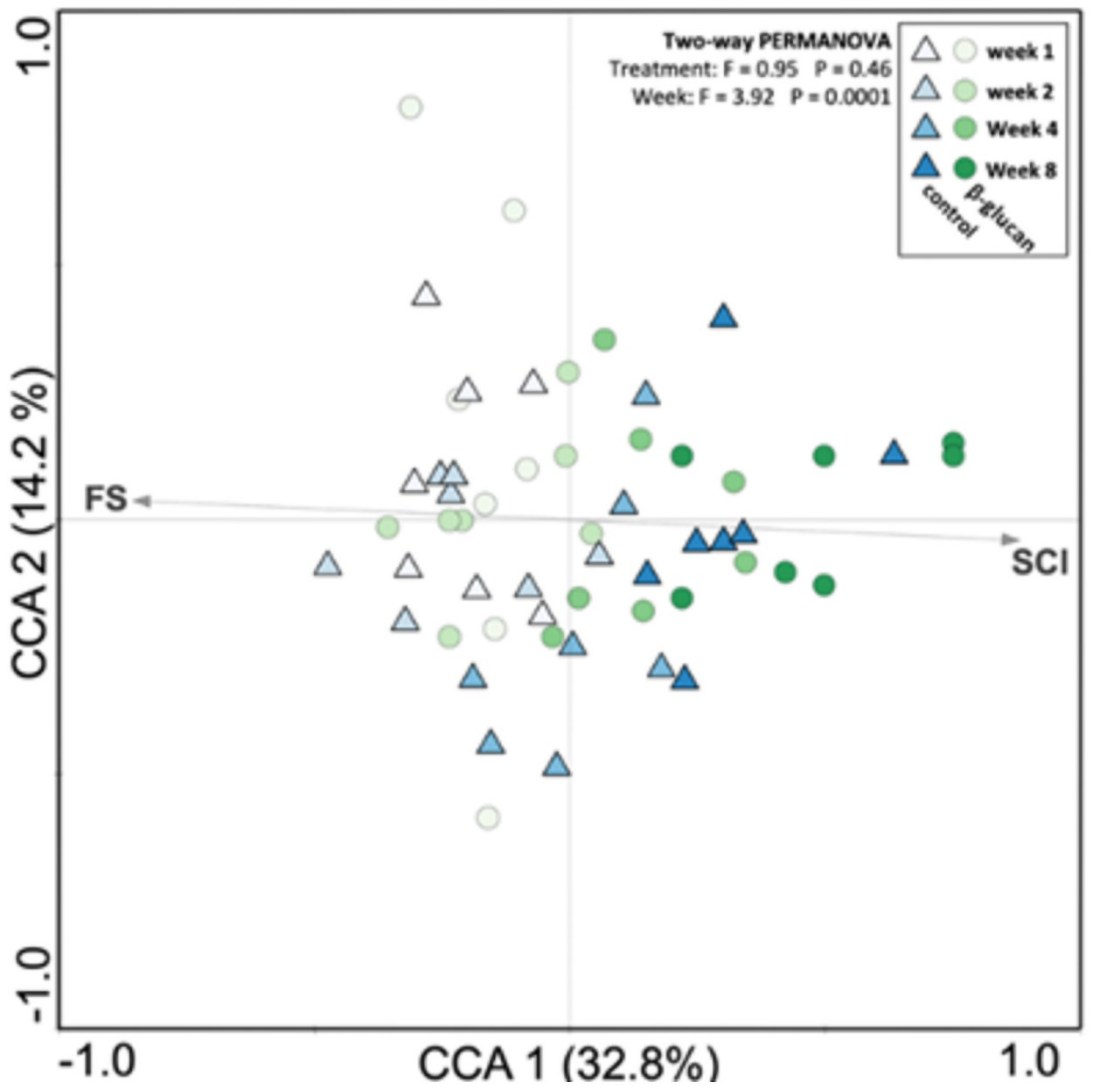
Correlation between dairy calves’ starter concentrate intake (SCI) and fecal score (FS) with bacterial structure. Canonical correspondence analysis of the bacterial community patterns and these two variables. Arrows indicate correlation between SCI/FS and microbial profile. Only significant parameters factor is shown.

The general association of the bacteriome data with performance and animal health data shows that certain parameters were significantly correlated to the bacterial community structure (Fig 6). The starter concentrate intake (SCI) had a significant correlation to the bacterial community at week 8, while the fecal score (FS) had a high correlation to the bacterial community at weeks 1 and 2.

**Fig 7:**
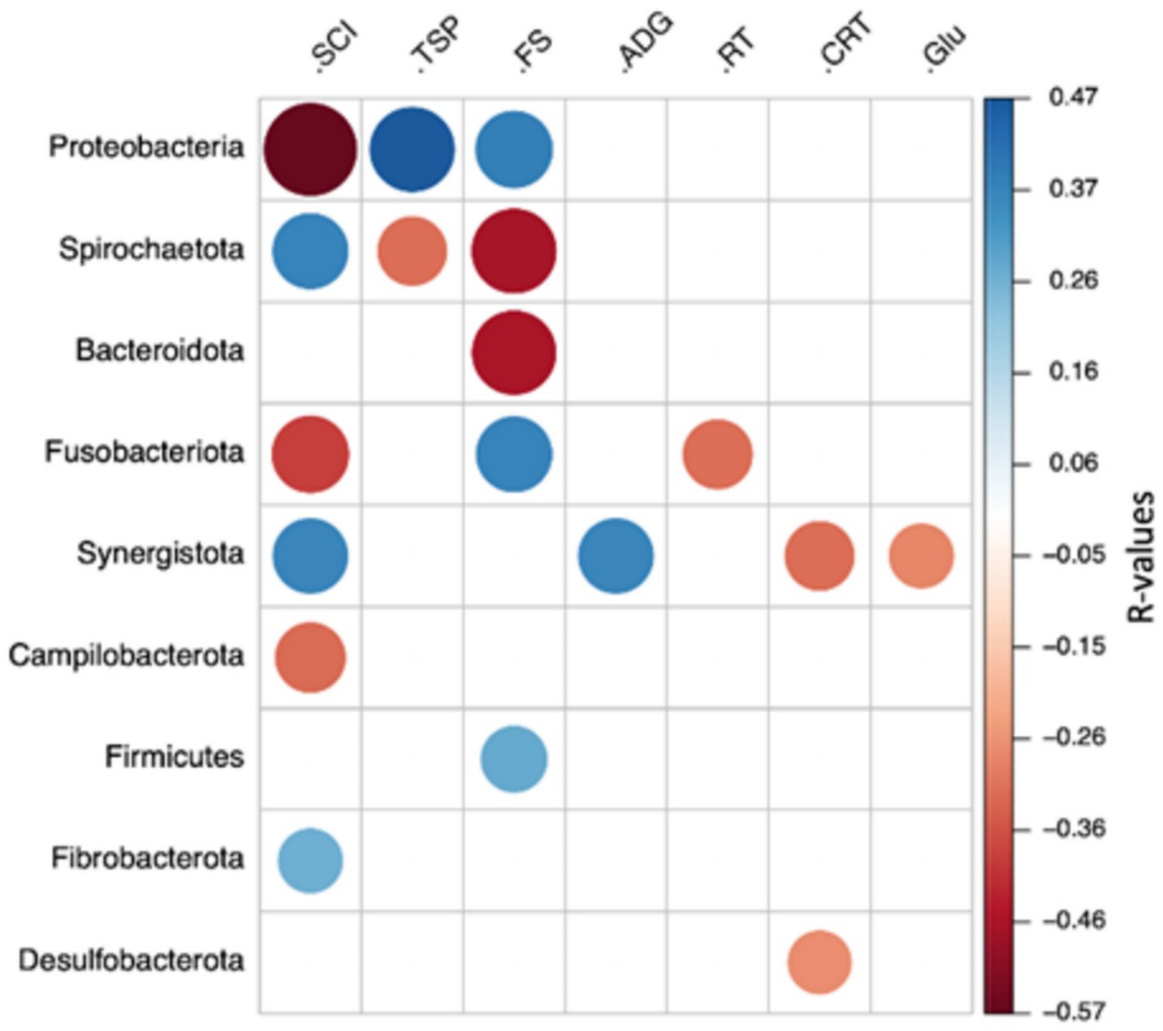
Heatmap showing the Spearman’s rank correlation coefficients and statistical significance between phyla abundance and calves’ parameters. Blue and red colors indicate significant positive and negative correlations, respectively (*P* < 0.05). SCI, starter concentrate intake; TSP, total serum protein; FS, fecal score; ADG, average daily gain; RT, rectal temperature; CRT, creatinine; Glu, glucose.

Besides structure, the bacterial composition also correlated with certain animal performance, health, and metabolic parameters (Fig 7). The phyla Firmicutes, Proteobacteria, and Fusobacteriota had a positive correlation with the fecal score, while Bacteroidota had a negative correlation. Proteobacteria had a high negative correlation with concentrate intake but a positive correlation with total serum protein (TSP).

**Fig 8:**
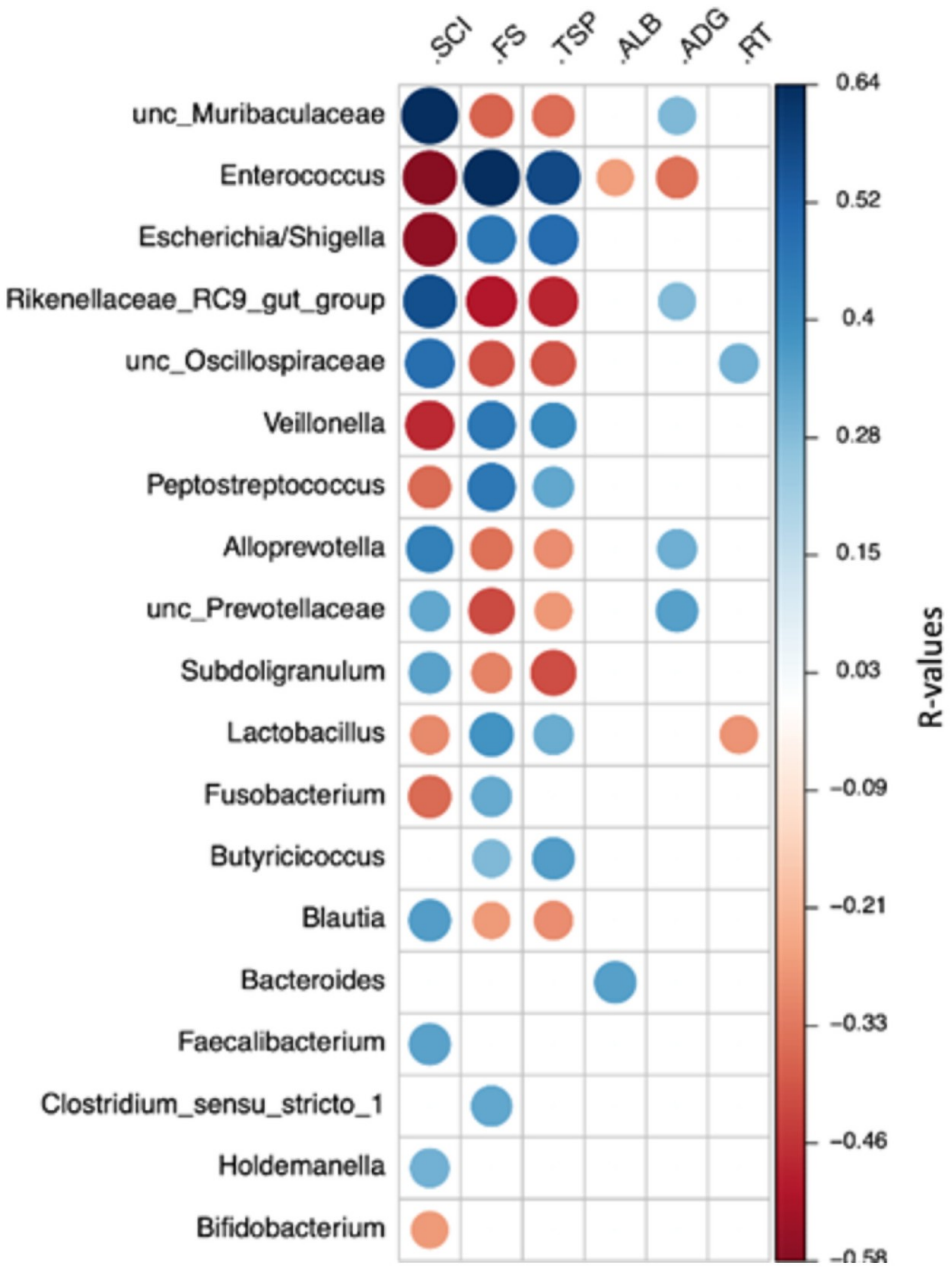
Heatmap showing the Spearman’s rank correlation coefficients and statistical significance between genera abundance and calves’ parameters. Blue and red colors indicate significant positive and negative correlations, respectively (*P* < 0.05). SCI, starter concentrate intake; FS, fecal score; TSP, total serum protein; ALB, albumin; ADG, average daily gain; RT, rectal temperature.

Those genera that showed a negative correlation with the SCI had a positive correlation with the FS, and vice versa (Fig 8). Other interesting points were the positive correlation of albumin with *Bacteroides* and negative with *Enterococcus*, the negative correlation of *Lactobacillus* to rectal temperature (RT), and the positive correlation of *Alloprevotella* to average daily gain (ADG). The genera presented in this figure had an abundance ≥ 1% and all correlations are in S5 Table.

## Discussion

β-glucans have a direct effect on the human and animal immune system [1], and stimulate gut microbiota activity in the lower gastrointestinal tract. The effect on the gut microbiome has been observed mostly in vitro and in human [6, 8] and pigs [7, 9]. However, in our study, we observed no effect on bacterial diversity and structure. Probably the amount of BG supplemented was not enough to affect the fecal microbial structure. According to Joyce et al. [34], oat BG has the greatest effect on the gut microbiota and Singh et al. [1] stated that the higher molecular weight, the more efficient BG is. However, no studies have described the effect of different sources or molecular weight of BG on the bacterial community of dairy calves.

Although no differences were found in bacterial community structure and diversity, we found an increased abundance of the genera *Alloprevotella* and *Holdemanella* associated with BG supplementation. The genus *Alloprevotella* has an anti-inflammatory function [35], produces succinate and acetate and is associated with the improvement of the intestinal barrier [35, 36]. Interestingly, this genus also showed a positive correlation with ADG, suggesting that BG supplementation may be a way to change *Alloprevotella* abundance, which improves performance. In a recent study by Reis et al. [17], it was shown that BG supplementation led to heavier weaned calves at 56 days (56.35 vs 51.53 kg) and they tended to be more efficient than control (0.29 vs 0.24) during the pre-weaning period. The genus *Holdemanella* is associated with high-fat diets and also related to lipid metabolism [37]. The milk replacer used in our work contains in its composition two oils extracted from vegetable sources [15]. However, Bittar et al. [38] stated that the digestive system of growing calves might not be fully developed to digest ingredients of vegetable sources in early ages. Based on this, BG supplementation possibly stimulated the growth of this bacterial genus to assist in the metabolism of the vegetable oils included in the milk replacer.

The most significant differences in the fecal bacterial community were associated with age. Increasing diversity and dissimilarities among weeks have been observed in other studies and were associated with calf growth and development, as well as increasing starter concentrate intake [15, 39, 40]. In our data, the concentrate intake was more correlated with the bacterial community at week 8 of calves’ life.

The starter concentrate intake, besides being essential for nutrient supply and rumen development, was shown to correlate positively with the bacterial genera *Blautia*, *Alloprevotella*, and *Faecalibacterium*. Hennessy et al. [41] also observed an increased abundance of *Blautia* as animals made the transition from liquid to a solid diet. In addition, these authors have noted that the genus *Faecalibacterium* has a positive correlation with *Blautia*. According to Dias et al. [42], *Blautia*, similar to *Alloprevotella*, also produces acetate by fermentation of carbohydrates, and *Faecalibacterium* uses this acetate as a substrate for growing. The negative correlation of *Bifidobacterium* and *Lactobacillus* with starter concentrate intake may be associated with the competition with other carbohydrate-degrading bacteria [15, 42].

Besides the starter concentrate intake, the bacterial community structure in the initial weeks (1 and 2) was correlated with fecal score. This may be mainly related to the genus *Escherichia/Shigella*, which correlates positively with the fecal score. *Escherichia/Shigella* is generally associated with neonatal diarrhea cases and is abundant during the first two weeks of life [15, 43]. The bacterial genera *Fusobacterium* and *Clostridium* are both associated with bowel inflammation and were abundant during the period of the higher fecal scores [15, 44]. It is worth noting that *Lactobacillus* is also positively related to fecal score and TSP, and negatively related to rectal temperature, which may indicate that this genus is abundant in episodes of diarrhea, supporting and minimizing the effects of a possible infection. *Veillonella* and *Enterococcus*, other beneficial microorganisms, were also positively correlated with fecal scores, perhaps minimizing the effects of diarrhea. These bacterial genera have been associated with the fecal microbiota of healthy calves and may be considered as a probiotic with a bacteriocin-producing ability [45, 46].

Although we observed a high abundance of the genus *Collinsella*, which is associated with pro-inflammatory dysbiosis [47], no correlation was found with the fecal score, probably due to the high abundance throughout the pre-weaning phase. BG supplemented calves had lower fecal scores (1.06 vs 1.46) and consequently a reduced period of diarrhea (14.7 vs 25.7 d) [17]. BG can potentially decrease the attachment of pathogens to the intestinal epithelium, preventing inflammatory processes [4]. Thus, feeding BG can have important effects on calf health and performance.

## Conclusions

Taken together, our study showed that algae β-glucan have a small effect on the fecal bacteriome. However, despite the minor effect observed, the increased abundance of *Alloprevotella* and *Holdemanella* indicates a beneficial effect of β-glucans supplementation on dairy calves.

## Supporting information

S1 Table Sequencing information. (XLSX)

S2 Table ASV identification of samples collected in dairy calves’ feces fed milk replacer supplemented or not with β-glucans. #N/D: not detected.
(XLSX)

S3 Table Abundance of phyla over the weeks in dairy calves’ feces fed milk replacer supplemented or not with β-glucans. (XLSX)

S4 Table Abundance of genera over the weeks in dairy calves’ feces fed milk replacer supplemented or not with β-glucans. #N/D: not detected.
(XLSX)

S5 Table Correlation coefficients phyla and genus abundance and calves’ parameters. (XLSX)
